# Dairy food supplementation may reduce malnutrition risk in institutionalised elderly

**DOI:** 10.1017/S000711451600461X

**Published:** 2017-01-18

**Authors:** Sandra Iuliano, Shirley Poon, Xiaofang Wang, Minh Bui, Ego Seeman

**Affiliations:** 1 Department of Endocrinology/Medicine, University of Melbourne/Austin Health, West Heidelberg, VIC 3081, Australia; 2 Melbourne School of Population & Global Health, University of Melbourne, Parkville, VIC 3010, Australia; 3 Institute of Health and Ageing, Australian Catholic University, Melbourne, VIC 3000, Australia

**Keywords:** Aged-care facilities, Dairy foods, Elderly, Malnutrition, Mini Nutrition Assessment

## Abstract

Malnutrition in institutionalised elderly increases morbidity and care costs. Meat and dairy foods are high-quality protein sources so adequate intakes may reduce malnutrition risk. We aimed to determine whether inadequate intakes of meat and dairy foods contribute to malnutrition in institutionalised elderly. This cross-sectional study involved 215 elderly residents (70·2 % females, mean age 85·8 years) from twenty-one aged-care facilities in Melbourne, Australia. Dietary intake was assessed using observed plate waste. Food groups and serving sizes were based on the Australian Guide to Healthy Eating. Nutrient content was analysed using a computerised nutrient analysis software (Xyris). Malnutrition risk was assessed using the Mini Nutrition Assessment (MNA) tool; a score between 24 and 30 indicates normal nutritional status. Data were analysed using robust regression. Mean MNA score was 21·6 (sd 2·7). In total, 68 % of residents were malnourished or at risk of malnutrition (MNA score≤23·5). Protein intake was 87 (sd 28) % of the Australian recommended dietary intake (RDI). Consumption averaged 1 serving each of dairy foods and meat daily. Number of dairy and meat servings related to proportion of protein RDI (both *P*<0·001), with the former contributing 13 % and the latter 12 % to protein RDI. Number of dairy servings (*P*<0·001), but not meat servings increased MNA score; each dairy serving was associated with a 1 point increase in MNA score so based on current intakes, on average if residents consumed the recommend four dairy servings (addition of 3 points to MNA score) they would achieve normal nutrition status (>24 points). Provision of meat and dairy foods did not meet recommended levels. On the basis of current dietary intakes in aged-care residents, increasing consumption of dairy foods to the recommended four servings daily ensures protein adequacy and may reduce malnutrition risk in institutionalised elderly, and so reduce risk of comorbidities and costs associated with malnutrition.

Malnutrition in institutionalised elderly is endemic, with up to 89 % of residents being at risk of malnutrition or malnourished^(^ ^1^ ^)^. Malnutrition is associated with higher morbidity and poorer health outcomes^(^ ^2^ ^)^. Risk factors for malnutrition in institutionalised elderly include age, sex (female), cognitive impairment, level of dependency, swallowing difficulties and not consuming all foods offered^(^ ^3^ ^)^. In Australia the majority of institutionalised elderly are women over 80 years of age.

The annual cost of malnutrition in institutionalised elderly in the Netherlands is estimated at €8000 (approximately US$9000) for each resident at risk of malnutrition and €12 000 (approximately US$13 000) for each malnourished resident^(^ ^4^ ^)^. Similar analyses are not available in Australia, however in the hospital setting, after controlling for underlying conditions and treatments, malnutrition added about AU$1800 (approximately €1200 or US$1300) to each admission^(^ ^5^ ^)^. As the population ages, demand for institutionalised care will increase, and so too will the burden of poor health related to malnutrition in the elderly.

Institutionalised elderly are predominantly reliant on aged-care providers to meet their nutritional needs. Nearly all food is provided through the facility’s food service, with most residents having limited access to externally prepared foods. These circumstances necessitate that aged-care providers offer nutritionally adequate meals and snacks. Despite nationally available guidelines and supervision by dietitians to assist staff with menu planning, a study of fourteen aged-care facilities revealed that foods provided do not always meet nutritional requirements, with protein intakes frequently below recommended^(^ ^6^ ^)^. Meat and alternatives (poultry, fish, eggs, tofu, nuts, seeds, legumes/beans) and dairy foods (milk, yogurt, cheese) provide protein and assist in meeting protein requirements with adequate provision and consumption^(^ ^7^ ^)^. Therefore we hypothesised that in aged-care residents, inadequate intakes of meat and dairy foods would be associated with protein insufficiency and increased risk of malnutrition.

## Methods

Data were collected from a convenience sample of ambulant elderly (>10 % of all residents) from twenty-one aged-care facilities in metropolitan Melbourne and regional Victoria, Australia, between November 2013 and May 2014 as part of baseline assessments for a large cluster-randomised placebo-controlled trial. Inclusion criteria for the trial were: (i) facilities required accreditation by the Australian Aged-Care Quality Agency, and (ii) they accommodated ambulant residents. Written consent was obtained from residents, or next of kin to monitor dietary intake and perform nutrition assessments. Residents provided written consent to perform blood sampling. The overall study was approved by the Austin Hospital Human Research Ethics committee (project number 04958) and is registered on the Australian New Zealand Clinical Trials Registry (ACTRN12613000228785).

Food service at facilities operate cook-fresh systems, with all foods prepared on-site on the basis of 4-week rotating menus. Typical meal service consisted of a continental-style breakfast (occasional hot breakfast), a mid-day meal providing a hot dish and dessert, an evening meal consisting of soup and choice of a hot or cold dish and dessert, and morning, afternoon and evening snacks.

Trained dietitians determined dietary intake on two random days using the validated method of visual estimation of plate waste^(^ ^8^ ^)^. Standard serves were weighed on a digital food scale (±1 g) (Soehnle Page Profi), and foods and beverages served and wasted were compared against the standard serve using a seven-point scale. The seven-point scale represents portions of each food consumed (or remaining): 0=no food remaining, +M=1 mouthful remaining, 1/4=25% remaining, 1/2=50% remaining, 3/4=75% remaining, −M=1 mouthful consumed (90% remaining), 1=no food eaten. Meals served were rated against the standard meal (medium given the value of 100 %); small serving=75 %, large serving=125 %, extra large serving=150 %. Consumption was calculated as the difference between amounts served and wasted.

Foods were categorised into one of the five recommended food groups: vegetables and legumes/beans; fruits; grain (cereal) foods; lean meat, poultry, fish, eggs, tofu, nuts/seeds, legumes/beans (termed ‘meat’); and milk, yogurt, cheese and/or alternatives (termed ‘dairy’)^(^ ^9^ ^)^. On the basis of Australian Guide to Healthy Eating, if legumes/beans are the protein source (e.g. vegetarian dish), it is classified as ‘meat’, but in the presence of ‘meat’ it is classified as a ‘vegetable’^(^ ^9^ ^)^. Legumes/beans made up only 0·44 % of all foods consumed (data not shown), and were eaten during meals containing meat, further these were included in the ‘vegetables and legumes/beans’ food group. Foods not fitting into the five food groups were considered ‘discretionary’, and these were foods high in energy, saturated fats, added sugars, added salt, or alcohol, for example cakes, biscuits, pastries, spreads, soft drinks, butter, cream, ice cream and processed meats^(^ ^9^ ^)^. Mixed dishes not considered ‘discretionary’ were analysed by categorising each ingredient into one of the five food groups.

Nutrient intakes were calculated using a computerised nutritional analysis software (Xyris Software (Australia) Pty Ltd) using product-specific nutritional information on packaging. Where packaging information was not available, nutrient values were derived from NUTTAB 2010^(^ ^10^ ^)^. There were no missing values for energy or protein.

Energy requirements were estimated on the basis of Schofield equation using estimated height from ulna length and applying a physical activity level of 1·20^(^ ^11^ ^)^. Protein requirements were based on age- and sex-specific recommended dietary intakes (RDI); males >70 years, 1·07 g/kg body weight (BW); women >70 years, 0·94 g/kg BW^(^ ^12^ ^)^.

Nutritional status was assessed by a qualified dietitian using the Mini Nutritional Assessment (MNA), a validate tool that consist of eighteen questions related to nutritional status, with a maximum score of 30^(^ ^13^ ^)^. Malnutrition was defined as a score <17; at risk of malnutrition for scores between 17 and 23·5; and normal nutritional status for scores between 24 and 30^(^ ^13^ ^)^. When required, nursing or care staff assisted with responses, and objective measures, for example weight changes, obtained from medical records maintained at facilities.

A sub-sample of ninety-two females and thirty-five males underwent morning fasting blood tests analysed for albumin (Cobas 701; Roche), Hb (Sysmex XN20 analyzer; Roche), insulin-like growth factor-1 (IGF-1) (LIAISON), 25-hydroxy vitamin D (25(OH)D) and parathyroid hormone (PTH) (immunochemistry, Cobas E170; Roche).

Distribution of variables was checked for normality using Shapiro–Wilk test. Sex comparisons were conducted using two-sample independent *t* test for normally distributed variables otherwise nonparametric Mann–Whitney test was used. For a categorical variable Fisher’s exact test was used. The association between the outcome variables, malnutrition risk (MNA score) or proportion of RDI (% RDI) for protein and predictors (number of meat or dairy servings), were examined using robust regression. For univariate analyses data were adjusted for sex, age and weight, and for multivariate analyses predictors were also adjusted for each other. All analyses were carried out using Stata 13.1 software (StataCorp LP).

## Results

Data were collected from 215 ambulant residents (70 % females, mean age 85·8 (sd 7·5) years). In total, 208 residents were able to self-feed, seven required feeding assistance; 211 residents consumed regular foods, four required texture-modified diets. On the basis of MNA scores, 68 % of residents were malnourished or at risk of malnutrition and mean protein intake provided 87 (sd 28) % of the RDI (Table 1). Mean albumin (36 (sd 4) g/l), Hb (128 (sd 16) g/l), IGF-1 (16 (sd 6) nmol/l) and 25(OH)D (75 (sd 27) nmol/l) were within the normal reference ranges, whereas mean PTH (6·8 (sd 4·1) pmol/l) was on the upper end of the normal range of 1·6–6·9 pmol/l (Table 1). Albumin levels were positively related to MNA score (*r* 0·25, *P*=0·005), but no relationship was observed between IGF-1 and MNA score (*r* 0·00, *P*=0·995). MNA score was related to number of medical conditions (*r* −0·14, *P*=0·039), but not number of medications (*r* −0·11, *P*=0·132). Each additional medical condition was associated with a 0·14 lower MNA score. As secondary indicators of nutritional adequacy mean intakes of Fe (men 9·3 (sd 3·8) mg/d; women 8·1 (sd 2·6) mg/d) met the RDI (8 mg/d for both sexes), but intakes for Zn (men 7·8 (sd 2·9) mg/d; women 6·6 (sd 1·9) mg/d) were below the RDI of 14 and 8 mg/d, respectively.

**Table 1:** Baseline characteristics, and comparison between elderly males and female aged-care residents† (Mean values and standard deviations)

	All (*n* 215)		Female (*n* 151)		Male (*n* 64)		
	Mean	sd	Mean	sd	Mean	sd	*P*
Age (years)	85·8	7·5	86·5	6·6	84·0	8·9	0·110**
Height (m)	1·64	0·08	1·60	0·05	1·73	0·05	<0·001
Weight (kg)	68·9	15·7	65·3	14·3	77·5	15·6	<0·001**
BMI (kg/m^2^)	25·6	5·3	25·5	5·5	25·9	4·8	0·624
Medications (*n*)	11·0	4·0	12·0	4·0	11·0	4·0	0·129
Medical conditions (*n*)	10·0	4·0	10·0	4·0	10·0	5·0	0·960
Albumin (g/l)	36·0	4·0	36·0	4·0	37·0	3·0	0·438*
Hb (g/l)	128·0	16·0	125·0	15·0	135·0	15·0	<0·001*
IGF-1 (nmol/l)	16·0	6·0	16·0	6·0	15·0	6·0	0·435*
25(OH)D (nmol/l)‡ §	75·0	27·0	78·0	26·0	67·0	29·0	0·032*
PTH (pmol/l)‡	6·8	4·1	6·5	4·2	7·4	4·0	0·173**
MNA score	21·6	3·7	21·7	3·8	21·6	3·5	0·656**
Normal/at risk/malnourished (%)	32/57/11		31/57/12		34/58/8		0·689***
Energy intake							
kJ/d	6307	1514	5973	1255	7075	1770	<0·001
kcal/d	1507	361	1427	300	1690	423	<0·001
% Estimated energy requirement	85	19	87	19	81	20	0·068
Protein intake							
g/d	56·0	16·0	54·0	14·0	60·0	20·0	0·040**
% RDI	87·0	28·0	91·0	28·0	76·0	24·0	<0·001**
Per kg body weight	0·8	0·3	0·9	0·3	0·8	0·2	0·082
Dietary Ca (mg/d)	622	263	612	241	647	317	0·852**

IGF-1, insulin-like growth factor-1; 25(OH)D, 25-hydroxy vitamin D; PTH, parathyroid hormone; MNA, Mini Nutrition Assessment. *P*-value computed using * *t* test, ** Mann–Whitney test and *** Fisher’s exact test for sex comparisons.

† Reference ranges: albumin: 32–43 g/l; Hb: 125–175 g/l; IGF-1: 6–27 nmol/l; 25(OH)D: 50–250 nmol/l; PTH: 1·6–6·9 pmol/l.

‡ *n* 92 females and 35 males.

§ In Australia mandatory fortification with vitamin D only occurs in edible oil spreads; 70 % of residents were administered a vitamin D supplement during data collection.

Some sex differences were observed (Table 1). Males were taller and heavier (but did not differ in BMI), consumed more protein daily, but achieved a lower proportion of their protein RDI than women. Men also tended towards meeting less of their estimated energy requirement, and consumed less protein/kg BW than women. Mean 25(OH)D was lower in males than females, and mean PTH in males was above the normal range (1·6–6·9 pmol/l). Mean Hb levels were within normal levels for both sexes relative to their sex-specific ranges. Males and females did not differ in the number of medical conditions or prescribed medications.

Mean intake of meats and dairy were 1·0 (sd 0·6) and 1·1 (sd 0·7) servings/d, respectively, both below the recommended intake levels of 2 and 4 servings/d for older women and 2·5 and 3·5 servings/d for older men, respectively^(^ ^9^ ^)^. Mean serves provided, wasted and consumed of each food group are presented in Table 2. Mean servings of meats and dairy foods provided did not meet the recommended levels. Food waste for meat was 0·3 (sd 0·3) (20·5 (sd 24·0) %) servings and 0·2 (sd 0·3) (13·9 (sd 17·7) %) servings for dairy.

**Table 2:** Mean number of serves provided, wasted and consumed daily by 215 elderly residents from twenty-one aged-care facilities (Mean values and standard deviations)

			Provided		Wasted		Consumed	
Food groups	Recommendations >70 years old		Mean	sd	Mean	sd	Mean	sd
Vegetables	Male	5	3·6	1·9	0·7	1·0	2·9	1·5
	Female	5	3·3	1·3	0·8	0·7	2·5	1·2
Fruits	Male	2	2·2	1·1	0·4	0·4	1·8	1·1
	Female	2	1·8	0·9	0·3	0·4	1·6	0·8
Grains (cereals)	Male	4·5	3·4	1·6	0·5	0·6	2·9	1·6
	Female	3	3·0	1·2	0·6	0·6	2·4	1·0
Meat	Male	2·5	1·4	0·7	0·2	0·3	1·2	0·7
	Female	2	1·2	0·6	0·3	0·4	0·9	0·5
Dairy	Male	3·5	1·3	0·7	0·1	0·2	1·1	0·7
	Female	4	1·3	0·7	0·2	0·3	1·1	0·6
Discretionary	Male	0–2·5	6·9	2·8	0·8	0·8	6·0	2·7
	Female	0–2	5·8	2·1	1·0	1·1	4·8	1·7

Serving sizes; meat: 65 g cooked lean meat, 80 g cooked lean poultry, 100 g cooked fish fillet, two large eggs, one cup cooked/canned legumes, 170 g tofu, 30 g nuts or seeds; dairy foods: 250 ml milk, 200 g yogurt, 40 g cheese, 1/2 cup evaporated milk or ricotta cheese; vegetables: 1/2 cup cooked vegetables, sweet corn or cooked, dried or canned beans, peas, lentils, one cup green leafy or raw salad vegetable, 1/2 potato or starchy vegetable, one medium tomato; fruits: one medium or two small fruits, one cup diced or canned fruits; grains: one slice bread, 1/2 bread roll, 1/2 cup cooked, pasta, rice, noodles, porridge, 2/3 cup cereal flakes, 1/4 cup muesli, three crispbreads, one crumpet, one English muffin or scone; discretionary: two scoops ice cream, two slices processed meats, two thin sausages, two to three sweet biscuits, one doughnut, five to six lollies, one small slice of cake, two tablespoons honey or jam, 1/2 small bar of chocolate, two tablespoons cream, one tablespoon margarine or butter, 1/3 commercial meat pie, twelve fried chips, two standard alcoholic drinks^(^ ^9^ ^)^.

For both univariate and multivariate (adjusted for age, sex and weight) regression analyses number of dairy and meat servings consumed was associated with proportion of protein RDI met (*P*<0·001). Each dairy serving contributed 13 % and meat serving 12 % protein RDI, so on average residents would achieve 100 and 99 % of the RDI for protein, respectively, with an additional dairy or meat serving above current consumption (Table 3). Univariate analysis indicated only number of dairy servings (*P*=0·004) was associated with MNA score (Table 3). Each dairy serving contributed 1 point on the MNA score, so within the context of their current dietary intake, if residents consumed the recommended four dairy servings daily they would, on average, achieve normal nutritional status (MNA score >24) and only 1/3 of residents would be classified as malnourished or at risk of malnutrition (data not shown). Including number of medical conditions or those requiring feeding assistance or a texture-modified diet to the models did not alter outcomes.

**Table 3:** Robust regression fitted to the data to examine the relationship between malnutrition risk score (MNA) or proportion of recommended protein intake (% RDI) and dairy and meat consumed by elderly aged-care residents

		Univariate			Multivariate		
Outcome	Predictor	Coefficient	se	*P*	Coefficient	se	*P*
MNA	Dairy serves eaten	1·05	0·36	0·004			
	Meat serves eaten	0·45	0·43	0·296			
% RDI	Dairy serves eaten	12·5	2·18	<0·001	13·2	1·98	<0·001
	Meat serves eaten	11·2	2·63	<0·001	12·1	2·36	<0·001

## Discussion

In all, 68 % of these institutionalised elderly were considered malnourished or at risk of malnutrition. Prevailing intakes of meats and dairy servings did not meet recommended levels. Consuming inadequate dairy servings contributed to risk of malnutrition and inadequate protein intake, whereas consuming insufficient meat servings influenced adequacy of protein intake. Provision of meats and dairy did not meet recommended levels.

The rates of malnutrition observed were similar to previous reports^(^ ^1^ ^)^. Malnutrition is associated with greater morbidity, so in addition to direct costs of malnutrition, indirect costs are likely, due to greater care needs, and protracted hospitalisation with more complications^(^ ^2^ ^)^. Preventing malnutrition will likely have cost-saving benefits for aged-care providers and the health system.

Mean protein intakes in residents were below recommended levels^(^ ^12^ ^)^. Protein malnutrition exacerbates aged-related loss of muscle mass and function, and adequate protein may prevent muscle loss^(^ ^14^ ^)^. Houston *et al*.^(^ ^15^ ^)^ observed in over 2000 elders (aged 70–79 years) that those in the highest quintile for energy-adjusted protein intake (approximately 1·1 g/kg BW per d) lost 40 % less total and appendicular lean mass over 3 years compared with those in the lowest quintile (approximately 0·7 g/kg BW per d).

Some investigators suggest that the recommended protein intake of 0·75–1·0 g/kg BW per d for the elderly may be insufficient to maintain lean mass, and intakes between 1·0 and 1·3 g/kg BW per d are required to maintain N balance in the elderly, because of age-related anabolic resistance and reduced protein synthesis efficiency^(^ ^12^ ^,^ ^16^ ^)^. For elderly with acute or chronic diseases, intakes of 1·2–1·5 g/kg BW per d are considered tolerable, except for those with end stage renal failure^(^ ^17^ ^)^. Inflammation, associated with illness, injury or disease increases energy requirement, impairs protein utilisation and increases N excretion thus increasing protein needs. For elderly in aged-care mild to moderate inflammation may be present due to chronic disease or illness and may increase protein requirements. van Nie-Visser *et al*.^(^ ^18^ ^)^ observed in nearly 20 000 aged-care residents that mean number of diseases was associated with malnutrition. We also observed an association between number of medical conditions and MNA score; however, in aged-care malnutrition may also relate to inadequate intake, so improving intake would likely improve nutritional status in residents^(^ ^6^ ^,^ ^19^ ^)^. Provision of meat and dairy did not meet recommended levels impeding the ability for residents to consume adequate dietary protein.

Both meat and dairy are sources of leucine, reported to increase IGF-1, muscle protein synthesis and lean muscle in the elderly, so consumption of these foods may help prevent age-related muscle loss^(^ ^20^ ^)^. Kramer *et al*.^(^ ^21^ ^)^ indicated that 21 g of whey protein (3 g of leucine) was sufficient to stimulate muscle protein synthesis in elderly (approximately 81 years of age) sarcopenic men. Low appendicular muscle is an indicator of poor nutritional status, which is captured in the MNA tool as calf and upper arm circumferences.

Although some authors suggest the provision of large amounts of protein in one main meal is sufficient to maximise muscle protein synthesis, others suggest protein intake should be evenly distributed throughout the day^(^ ^22^ ^,^ ^23^ ^)^. However, in contrast to these studies where protein intakes met or exceeded recommended levels, mean protein intake in our institutionalised elderly was inadequate, including leucine intake being below the suggested threshold to promote muscle protein synthesis. Correcting protein inadequacy may be required first before apparent benefits of protein distribution may be evident.

Residents consumed over 6 servings/d of discretionary foods, well in excess of the recommended 0–2 servings^(^ ^9^ ^)^. Consumption of discretionary foods in place of meat and dairy options, may limit the ability for residents to obtain sufficient protein, especially for those with reduced appetites^(^ ^6^ ^)^. Mila *et al*.^(^ ^7^ ^)^ observed in a sample of sixty-two Spanish nursing home residents, that with provision (and consumption) of adequate dairy and meat, residents met protein requirements. In contrast, Iuliano *et al*.^(^ ^19^ ^)^ reported that 61 % of 199 elderly from eighteen aged-care facilities consumed below recommended protein levels when consuming adequate meat, but inadequate dairy.

Strategies have been explored to improve protein intake and reduce malnutrition risk in institutionalised elderly. Meta-analyses of oral protein supplements (OPS) use in aged-care residents indicate weight gain of approximately 2·5 (95 % CI 1·7, 2·7) %, but few studies reported improvements to lean mass and physical function^(^ ^24^ ^)^. In aged-care OPS are often not delivered according to treatment plans and significant waste is observed^(^ ^25^ ^)^. Moreover, elderly with limited appetites may have difficulty in consuming supplements in addition to regular meals so normal intake may be compromised^(^ ^24^ ^)^. In a 6-week randomised pilot nutrition intervention, Simmons *et al*.^(^ ^26^ ^)^ observed a significant reduction in mealtime energy intake (−124 kcal/d, *P*<0·05) by eighteen nursing home residents when consuming supplements, resulting in no change to total intake. The authors observed a high rate of refusal of supplements raising uncertainty about long-term use of OPS to manage malnutrition.

Limited success with protein fortification of the food supply has been observed in aged-care. Grieger & Nowson^(^ ^27^ ^)^ investigated the provision of fortified milk in aged-care, and observed that without supervision and guidance, food service staff did not make sufficient menu modifications to increase milk intake, so no benefits were observed in nutritional status of residents. In an intervention trial in aged-care, Iuliano *et al*.^(^ ^28^ ^)^ reported that food service staff had difficulties mixing a dairy-based protein supplement into foods, limiting the quantity of protein provided. Pre-fortified products have been effective in improving nutritional status in institutionalised elderly but budgetary constraints may limit their use^(^ ^29^ ^)^. However, a cost-benefit analysis of enhanced dietary protein intake on malnutrition risk has not been undertaken, commercially available protein powders cost over four times that of protein from milk powder (approximately AU$8·00 *v.* <$2·00 for 100 g of protein), so may be feasible within limited budgets.

The provision of adequate meat and dairy foods is a reasonable approach to improving nutritional status of institutionalised elders. There are limited data on additional meat consumption to prevent malnutrition. A 50 % increase in muscle protein synthesis was observed in elders aged 68 (sd 2) years when consuming 113 (*n* 10) or 340 g (*n* 7) of beef^(^ ^30^ ^)^. Muscle protein synthesis was no greater with 340 g of beef therefore the authors suggested that meals containing about 30 g of protein (from meat) were sufficient^(^ ^30^ ^)^.

Improvements in nutritional intake were observed in sixty-eight aged-care residents when consuming two additional dairy servings daily^(^ ^31^ ^)^. Relative to residents consuming from regular menus (*n* 60), increased daily intakes were observed for energy (+900 kJ (215 kcal), *P*<0·001), protein (+25 g, *P*<0·0001) and proportion of estimated energy requirements (+18 %, *P*<0·0001). Protein and energy intakes remained below recommended levels in controls^(^ ^31^ ^)^. The long-term sustainability of menu changes to improve protein intake in institutionalised elders requires investigation.

The type of dairy food may also influence contribution of dairy, but not meat, to MNA score. Both meat and dairy are sources of quality protein; however, relative to MNA score, increasing milk intake contributes to both protein and fluid intakes, so each additional serving of milk has the capacity to add 1·5 points to the total MNA score. Moreover in line with dietary recommendations current meat intake in residents is approximately half the recommended two servings, so the addition of a meat serve will achieve recommended levels (but not alter MNA score) but dairy intake is approximately 25 %, with only one of the recommended four servings being consumed. Three additional dairy serves to current intake levels improves intake to recommended levels in line with the Australian Guide to Healthy Eating.

Food provision in aged-care in Australia is comparable between providers with similar menu cycles and foods offered, so two random days of intake data suitably represents food consumption in residents. Food groups and serving sizes were based on Australian standards, however, serving sizes are similar to other countries so translation of outcomes is reasonable.

In conclusion, rates of malnutrition in elderly in aged-care remain unacceptably high. Dietary strategies in aged-care should aim to improve protein intake and prevent malnutrition in residents. The provision of dairy foods, in line with recommended intake levels is likely a simple and cost-effective method that may reduce malnutrition risk in institutionalised elderly.
